# Geo-Analytic Decision Support for Federal Behavioral Health Prioritization Among American Indian and Alaska Native Populations: Exploratory Study

**DOI:** 10.2196/87265

**Published:** 2026-10-26

**Authors:** David M Paschane, Michael Ontiveros, John E Snyder

**Affiliations:** 1 Office of Clinical and Preventive Services, Indian Health Service, 5600 Fishers Lane, Rockville, MD, 20857, United States, 1 202-256-5763; 2 Albuquerque Area Southwest Tribal Epidemiology Center, Albuquerque Area Indian Health Board, Albuquerque, NM, United States; 3 Office of Planning, Analysis, and Evaluation, Health Resources and Services Administration, Rockville, MD, United States

**Keywords:** medical geography, decision-support, rural health, behavioral health, federal investments, American Indians, Alaska Natives, tribes, geographic information systems, GIS

## Abstract

**Background:**

Chronic diseases, including behavioral health topics, impose a significant burden on health systems, especially among rural and tribal communities. In response, federal agencies require reliable data-driven decision-support for investigating emerging needs, prioritizing responses, and conducting surveillance. Federal decision-support models may benefit from geo-analytic methods capable of integrating multicriteria evidence, interagency policy-driven parameters, and disparate jurisdictional interests.

**Objective:**

This study aims to identify candidate federal priorities for intergovernmental and interagency cooperation using exploratory geo-analytic decision-support frameworks, specifically related to behavioral health risks and services among tribal populations.

**Methods:**

We tested illustrative federal decision parameters within 3 exploratory geo-analytic frameworks using publicly available US data. These included alcohol-related and drug-related mortality data from 2018 to 2023 from the Centers for Disease Control and Prevention (CDC); facility locations, operating hours, and Euclidean-distance approximations of physical access to behavioral health services from the Indian Health Service (IHS); and federal transportation and rest stop data used to examine hypothesized interpersonal-risk conditions. Analyses were performed from February through August 2025.

**Results:**

The exploratory analyses identified 78 counties across 13 states as prioritized investigation areas based on elevated and increasing alcohol-related or drug-related death rates. The 6-year pooled crude death rates ranged from 1197.8 to 1848.7 per 100,000 American Indian or Alaska Native (AI/AN) persons. Two regional analyses demonstrated methods for further prioritization. One analysis estimated that 22,029 and 234,852 AI/AN persons resided in low physical access areas for services during typical and nontypical business hours, respectively. Another analysis identified 12 potential interpersonal risk-exposure locations within a region of 28 rest stop facilities among 80 AI/AN communities.

**Conclusions:**

Three exploratory geo-analytic frameworks demonstrate methodological feasibility for identifying candidate geographic priorities that could inform federal-tribal and interagency investigations, service access barriers, and interpersonal risk events. The recommendation is to validate and expand the methodology with rural and tribal systems, leading to a candidate federal decision-support model for future interagency initiatives.

## Introduction

In February 2025, Executive Order (EO) 14212 established a framework for refocusing federally funded scientific research, technology development, and health care delivery on mitigating the rising national rates of chronic disease [1]. The framework notes that 90% of the nation’s 4.5 trillion dollars in annual health care expenditures are for people with chronic diseases, including mental health conditions. The federal guidance calls for the nation’s health care systems to emphasize health promotion, not solely disease management.

Health care systems in the United States are largely based in urban areas, while 46 million Americans live in nonmetropolitan counties [2]. Rural communities experience greater barriers to accessing health services and higher morbidity and mortality rates from the nation’s leading causes of death [3]. Among these areas, American Indian or Alaska Native (AI/AN) persons engage with health systems operated by a mix of federally recognized tribes or tribal organizations, federal systems, and private rural systems. Consistently, AI/AN persons have lower life expectancy at birth, self-report high rates of fair or poor health (21.8%), and contend with significant burdens of heart disease, cancer, unintentional injuries, and chronic liver disease as leading causes of death [4]. Application of the new Health and Human Services (HHS) strategic framework for promoting health is therefore particularly germane to rural and tribal areas.

In this study, rural and tribal health systems are considered overlapping rather than mutually exclusive domains. Tribal members often reside within rural areas and may obtain care through a combination of tribal, federal, and other rural health systems. Their families and other members of the surrounding communities may also rely on the same regional health care infrastructure. Accordingly, rural and tribal areas are treated as combined areas of interest because service capacity, access constraints, and population health needs may extend across tribal and non-tribal jurisdictions. The principal analytic population in these demonstrations remains AI/AN persons; rural areas refer to the broader service geography in which many overlapping tribal, federal, and other health systems operate.

Health systems in rural and tribal communities tend to form and manage local and regional partnerships to achieve integrated service delivery and risk surveillance [5,6]. While such communities are often resilient and able to leverage insights that fit their unique contexts [7], they may be vulnerable to unexpected operational surges when complex health services are required. These cases tend to include the treatment of substance use disorders, violence-based injuries, mental illness, and debilitating chronic conditions [8]. Operational strain on local health systems can have broader social, economic, and safety consequences for communities. The ability to anticipate these surges and respond effectively is an essential rural health system capacity.

Relevant to both the HHS framework and the potentially overwhelming risks posed to rural and tribal communities, EO 14379 established the White House Great American Recovery Initiative [9]. The initiative enables federal agencies to explore and test decision-support capabilities in determining priorities in federal health service projects. Furthermore, EO 14332, Improving Oversight of Federal Grantmaking [10], encourages federal agencies to align discretionary awards with agency priorities and to establish clear benchmarks for measuring progress and success. Ideally, evidentiary methods should incorporate measurable necessity before issuing grants, as well as verify readiness for local and regional partnerships, opportunities for service innovations, and the soundness of proposed projects’ investments. The HHS framework and the related EOs provide an opportunity for federal agencies to reexamine their methods of analyzing and prioritizing programs and projects. Analytic methods must be comprehensive and repeatable to provide a reliable foundation for negotiations among federal agencies, tribes, and tribal organizations.

Nationally, there are an estimated 2.8 million AI/AN persons associated with 575 federally recognized AI/AN tribes [11,12]. Tribal governments lead community health concerns, including the unique federal-to-tribal requirements and opportunities to plan cooperative analyses and initiatives. Federal law establishes government-to-government responsibilities for tribal participation, consultation, self-determination, and, where applicable, self-governance in the administration of Indian health services [13,14]. This government-to-government context creates a need for reliable and verifiable federal decision-support methods. These are foundations for organizing and negotiating initiatives that affect tribes, tribal members, and their cooperation with other health system partners, including regional rural health systems.

Within the scope of chronic diseases, we opted to demonstrate our methods with behavioral health topics. Our baseline analysis focuses on national alcohol-related and drug-related (ADR) deaths, with a regional example of potential barriers to accessing behavioral health services. Substance use disorders may coexist with social and behavioral conditions associated with vulnerability to interpersonal victimization. Accordingly, the study also examines an exploratory geographic framework for identifying locations where hypothesized interpersonal risk conditions could be investigated. Potential severe interpersonal risks include stalking, assault, abduction, human trafficking, and homicide [15]. Since AI/AN communities are disproportionately impacted by human trafficking [16], the study also examines methodologies for planning surveillance with tribal and local authorities.

Geographic analysis can provide a common place-based framework for integrating evidence across jurisdictional interests. Herein, we explore the use of geographic sciences as an applicable set of decision-support methods for guiding the development of federal decision-support and surveillance tools. Because these methods rely on consistent location-based analyses, they are especially helpful in the context of coordinating strategic decision-making. These decisions involve actors operating across shared and overlapping geographies, including federal agencies, tribal governments, states, counties, and local health systems.

The field of geographic sciences includes place-based applied research for approximating useful patterns and anomalies, supporting hypothesis generation and, when combined with appropriate study designs, causal inference [17]. A subfield that is especially relevant to this study is medical geography [18]. This field specializes in the dynamic interactions among jurisdictional, economic, social, technological, political [19], and ecological factors [20]. These interactions inform measures of causal pathways in patient needs, community health needs [21], health system capabilities, and health outcomes [22]. Technologically, these methods are often performed through geographic information systems (GIS). GIS can provide efficiency in making repeated inquiries into multiple collocated or spatially diverse factors affecting health systems and patient experiences [23].

In combination, the GIS-based methods allow for multicriteria analyses that accommodate variability in both service objectives and local attributes that affect decisions among various interests [24]. The potential of geo-analytics in decision-support frameworks is the ability to foster a dynamic research community in governments, academia, and industry, leading to robust methods in support of complex, scalable projects [25]. The same community is raising concerns about the repeatability, reproducibility, and replicability of these projects [26]. The rapid interdisciplinary influence of advanced geo-analytics and medical geography, as well as the potential geospatial AI (GeoAI), has stimulated an interest in expanding scientific oversight. Areas of concern include the development of effective curricula for analysts and researchers [27], anticipating shortcomings in scientific methods and ethical controls [28], and sustaining data quality to reduce information bias [29].

This study had two objectives: (1) to identify candidate federal priorities for intergovernmental and interagency cooperation in strengthening behavioral health services among AI/AN populations within overlapping tribal and rural service environments and (2) to demonstrate reproducible GIS frameworks that could contribute to a federal decision-support model for health-risk surveillance and service planning.

## Methods

### Overview

This study developed and tested 3 geo-analytic frameworks (Figure 1) using geographic sciences to enable future analytic integration and decision-support modeling. We investigated patterns of elevated and increasing ADR mortality, physical barriers to services, and locations with hypothesized interpersonal risk conditions. For purposes of these exploratory analyses, we followed a form of administrative decision-making and selected illustrative thresholds to demonstrate how policy-relevant parameters could be incorporated into federal decision support. Federal investment decisions often reflect a combination of statutory, administrative, practical, and policy considerations, not solely empirical evidence [30]. Analyses were performed in GIS in R (version 4.1.2; R Foundation for Statistical Computing) [31].

**Figure 1.**
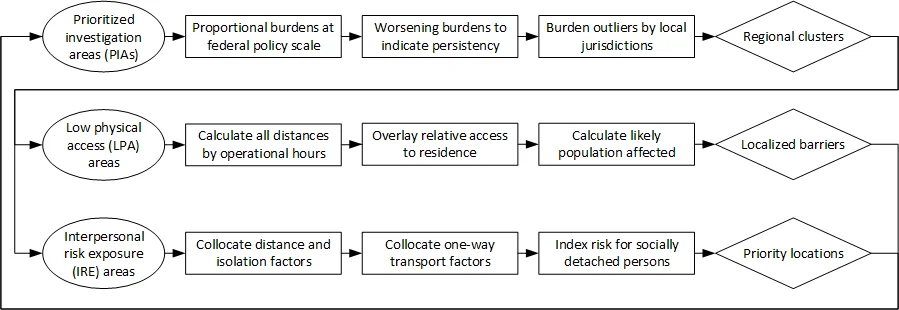
Conceptual structure of decision-support requirements, geo-analytic framework analyses, and initial analytic patterns to support the development of federal decision-support tools.

### National Prioritized Investigation Areas

The first geo-analytic decision framework enables the identification of prioritized investigation areas (PIAs, which are geographic areas that warrant prioritized investigation. We examined national ADR mortality data among AI/AN persons to locate PIAs based on geographically concentrated patterns of elevated and increasing mortality in AI/AN populations.

At the time of this analysis, we acquired 2018 to 2023 state-level and county-level mortality data from the Centers for Disease Control and Prevention (CDC) WONDER (Wide-Ranging Online Data for Epidemiological Research) database. We used the single race indicator for AI/AN, without restrictions. ADR deaths were identified using the National Center for Health Statistics Drug/Alcohol Induced Causes recode available in CDC WONDER. Analyses were restricted to deaths for which a drug-induced or alcohol-induced cause, as defined by the predefined *International Classification of Diseases, Tenth Revision (ICD-10)* [32] groupings, was listed as the underlying cause of death.

Using pooled crude mortality data from 2018 to 2023, we first identified states with greater than 1% of the aggregated death burden for the national AI/AN population. Within those states, we identified increases in death rates greater than 10% from 2018 to 2023. We then identified counties having 6-year pooled crude death rates exceeding the national non-AI/AN rate (958 deaths per 100,000 persons; 6-year pooled crude death rate) by 25%. The benchmark was calculated after excluding the count of AI/AN persons from the national rate. The national non-AI/AN rate was selected as an illustrative external benchmark for identifying AI/AN mortality burdens substantially exceeding the broader population burden; this comparator and the associated thresholds require sensitivity testing before operational use.

### Physical Access to Behavioral Health Services

The second geo-analytic decision framework facilitates potential characterization of local barriers to accessing health care. The analysis estimates physical proximity to behavioral health facilities serving AI/AN populations but does not determine individual eligibility for services at each facility. Eligibility may vary by facility, tribal affiliation, residence, and whether services are provided through Indian Health Service (IHS)–operated or tribally operated facilities. Accordingly, the measures represent potential geographic access rather than confirmed individual service eligibility.

To demonstrate this framework, we examined time and distance factors in accessing ambulatory and urgent/emergency behavioral health services for AI/AN populations in the American Southwest. The region focuses on Arizona and New Mexico, with portions of neighboring states that border them, including portions of California, Colorado, Nevada, and Utah. A December 2024 IHS national file of AI/AN-serving facilities [33], managed by either federal or tribal authorities [34], identified a sample of 119 facilities. We excluded schools and inpatient facilities from the analysis, resulting in 86 facilities classified as behavioral health services. Through online searches, we confirmed site data on the seasonality of weekly operating hours, health services offered, and latitude/longitude. Data representing 2 or more unique but collocated health services were disaggregated to maximize data quality on operating hours without impacting the precision of distance estimates. Service hours were measured as a component of typical weekday business hours (Monday through Friday, 8 AM to 5 PM) and nontypical business hours (Monday through Friday, 5:01 PM to 7:59 AM, plus Saturday and Sunday).

We created raster maps illustrating the entire landscape as a set of pixels. Raster cells represented 1 square mile, and each cell stored the Euclidean distance to the nearest eligible facility. The color changes at 10-mile intervals (≤10, >10‐20, >20‐30, and >30 miles). Using the raster maps as a reference for relevant distances across the surface, we produced choropleth maps to illustrate the region’s top 25% of census tracts by AI/AN population count. Population data are from the 2023 American Community Survey 5-year demographic and housing estimates [35]. The primary color code for the choropleth maps identifies census tracts completely within low physical access (LPA) areas, corresponding to locations greater than 30 miles from available services. The secondary color code identifies census tracts that were partial in these areas. All other areas, colored in white, represent tracts that were either beyond 30 miles of available services or excluded because they fell outside the top quartile by AI/AN population count.

### Anticipating Interpersonal Risk Exposure

The third geo-analytic decision framework is a proof of concept for identifying patterns of collocated factors as a potential interagency surveillance framework for informing human trafficking prevention. To demonstrate this approach, we analyzed collocated conditions representing hypothesized contextual conditions associated with interpersonal risk exposure (IRE). We selected Nevada for this exploratory analysis because we determined that it had the highest 2024 statewide per capita rate of reported human trafficking based on national hotline reports, which are supervised by federal agencies, at 7.2 cases per 100,000 people [36,37].

The exploratory analysis included a set of potentially interacting risk factors with weighted distance parameters as a candidate risk index measure. The parameters included straight-line (Euclidean) distances to major transportation routes, rest facility locations, and PIA borders. PIA proximity was included as an exploratory contextual indicator of home-area mortality burden, while transportation proximity represented potential mobility away from familiar social environments.

The framework assumes that vehicles traveling toward a major urban center may stop at rest facilities along the route. Conceptually, a rest facility may therefore serve as a point of confluence for possible victims and perpetrators [38,39]. The analysis is also informed by prior evidence linking mobility with changes in specific risk behaviors among some populations of people who use drugs [40]. Theoretically, transportation features may facilitate rural-to-urban mobility in circumstances in which people might be socially unaccompanied or detached, which the exploratory framework treats as a hypothesized context for possible exposure to interpersonal violence.

Data on rest facilities were accessed through the US Department of Transportation’s Bureau of Transportation Statistics, which identifies rest stops that accommodate commercial motor vehicles engaged in interstate transportation [41]. We acquired community locations and transportation routes for LPA and IRE analyses through the US Census 2023 TIGER (Topologically Integrated Geographic Encoding and Referencing)/Line Shapefiles [42].

For purposes of demonstration, we used 25 km (15.5 miles) as an initial distance of interest and weighted the factors according to a theoretical emphasis on proximity to a PIA and proximity to locations where exposure to exogenous risk is theoretically possible. These weights were selected a priori for proof-of-concept demonstration and were not empirically estimated or validated. The resulting score was calculated as follows:

IRE = 5 (PIA within 25 km) + 2 (route within 25 km) + 1.5 (rest facility within 25 km). A community receives the maximum score of 8.5 when it is within 25 km (15.5 miles) of all 3 features.

### Ethical Considerations

The Albuquerque Area Indian Health Board provided guidance on avoiding stigmatization, focusing on structural issues, and protecting Tribal data sovereignty. The IHS Institutional Review Board determined on July 1, 2026, that review was not required because the study used publicly available, aggregate data and did not constitute human-subjects research, as defined in 45 Code of Federal Regulations (CFR) § 46.102 [43]. The restriction of tribal identifiers was additionally used as an ethical safeguard against stigmatization [44]. Because applicable federal Indian health law requires active and meaningful tribal consultation for actions under the Indian Health Care Improvement Act (25 United States Code [USC] § 1602.5) [14], the decision parameters examined here are hypothetical and would require appropriate tribal consultation before operational adoption.

## Results

### National PIAs

Our analysis identified 20 states that represented at least 1% of the total ADR deaths among AI/AN persons from 2018 to 2023, ranging from 1.2% to 14.6%. We identified a subset of 14 states in which the 2023 ADR rate was at least 10% higher than the 2018 rate, as an indicator of increasing death burden. Percentage increases in ADR rates ranged from 11% to 36% for 14 states, representing 107,954 deaths over the 2018 to 2023 period. Within those 14 states, 78 counties met the PIA criterion of having a 6-year pooled crude ADR death rate at least 25% higher than the national non-AI/AN benchmark of 958 deaths per 100,000 persons. Across the 78 PIAs, 6-year pooled crude ADR death rates among AI/AN persons ranged from 1197.8 to 1848.7 per 100,000.

The distribution of the 78 PIAs (counties) was greatest in Oklahoma, with 17, followed by 14 in South Dakota, 13 in Alaska, 6 in Minnesota, and 6 in North Dakota. Arizona and North Carolina had 4 PIAs. Nevada and Washington had 3 PIAs. California, New Mexico, New York, and Oregon had 2 PIAs. One of the 14 states, Colorado, had no counties meeting this level of burden, or PIAs. There were 34,783 ADR deaths among the 78 PIAs, accounting for 32.2% (34,783/107,954) of the total sample of ADR deaths. Six PIAs accounted for 55.8% (19,400/34,783) of all ADR deaths among PIAs. Three Southwest PIAs accounted individually for 11.0% (3841/34,783), 11.9% (4133/34,783), and 12.5% (4339/34,783) of all ADR deaths occurring in the 78 PIAs. The remaining 3 of the 6 highest-burden PIAs were in South Dakota, Alaska, and North Carolina, with ADR deaths among all PIAs of 2.9% (1024/34,783), 6.1% (2106/34,783), and 11.4% (3957/34,783), respectively. Figure 2 identifies all 78 PIAs by states, illustrating apparent geographic concentrations of PIAs that cross state boundaries.

**Figure 2.**
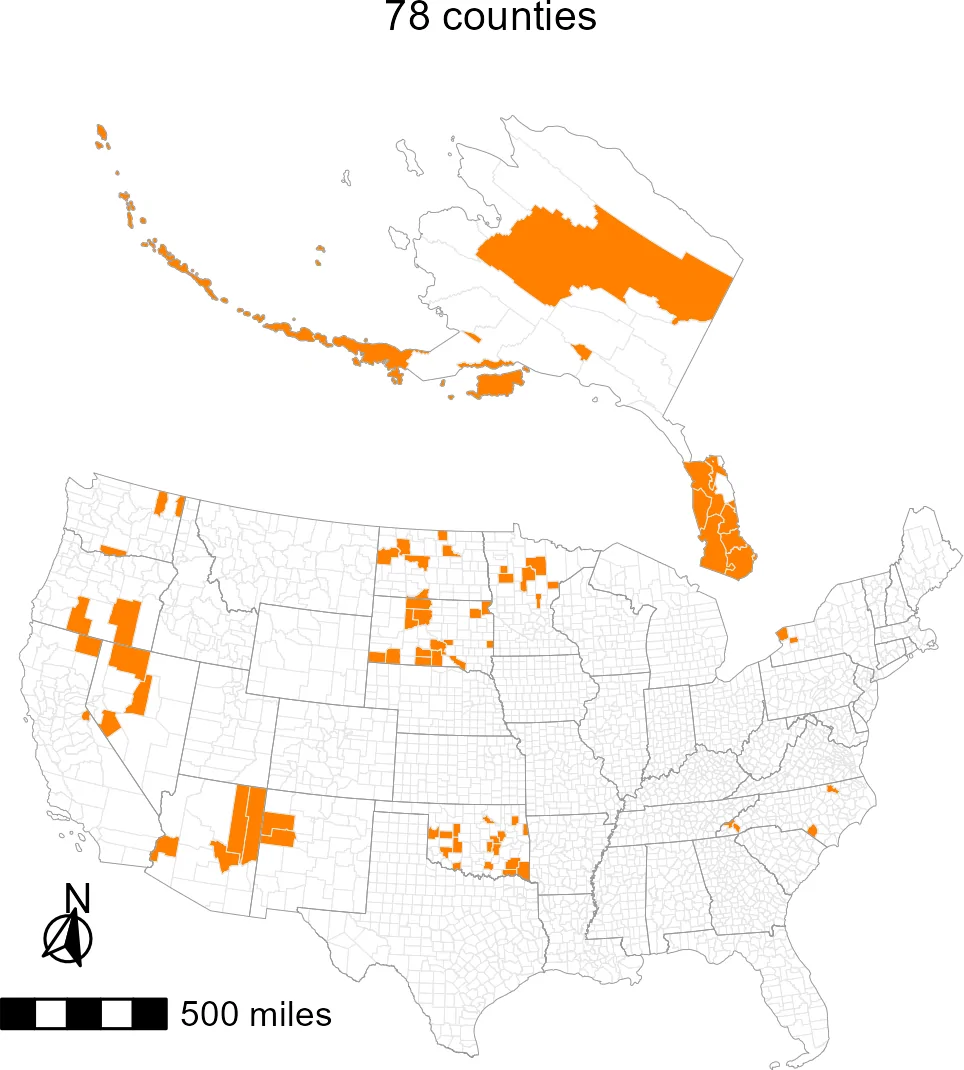
National distribution of prioritized investigation areas (PIAs) based on initial analyses of alcohol-related and drug-related death rates among American Indians and Alaska Natives.

### Physical Access to Behavioral Health Services

Concentrating on the example of the American Southwest, Figure 3 illustrates the distribution of ambulatory and emergency behavioral health service facilities and tribal lands. Figure 4 is a raster map of the ambulatory facilities operating during typical business hours. These depict straight-line distances from each facility, at 10-mile intervals, as an approximation of travel distances. Large areas beyond a 30-mile radius, representing LPA areas, are shown in red. Figure 5 identifies the distances to facilities operating during nontypical business hours. In this map, the area shown in red is much larger, indicating a substantially larger LPA area during nontypical business hours for AI/AN persons.

**Figure 3.**
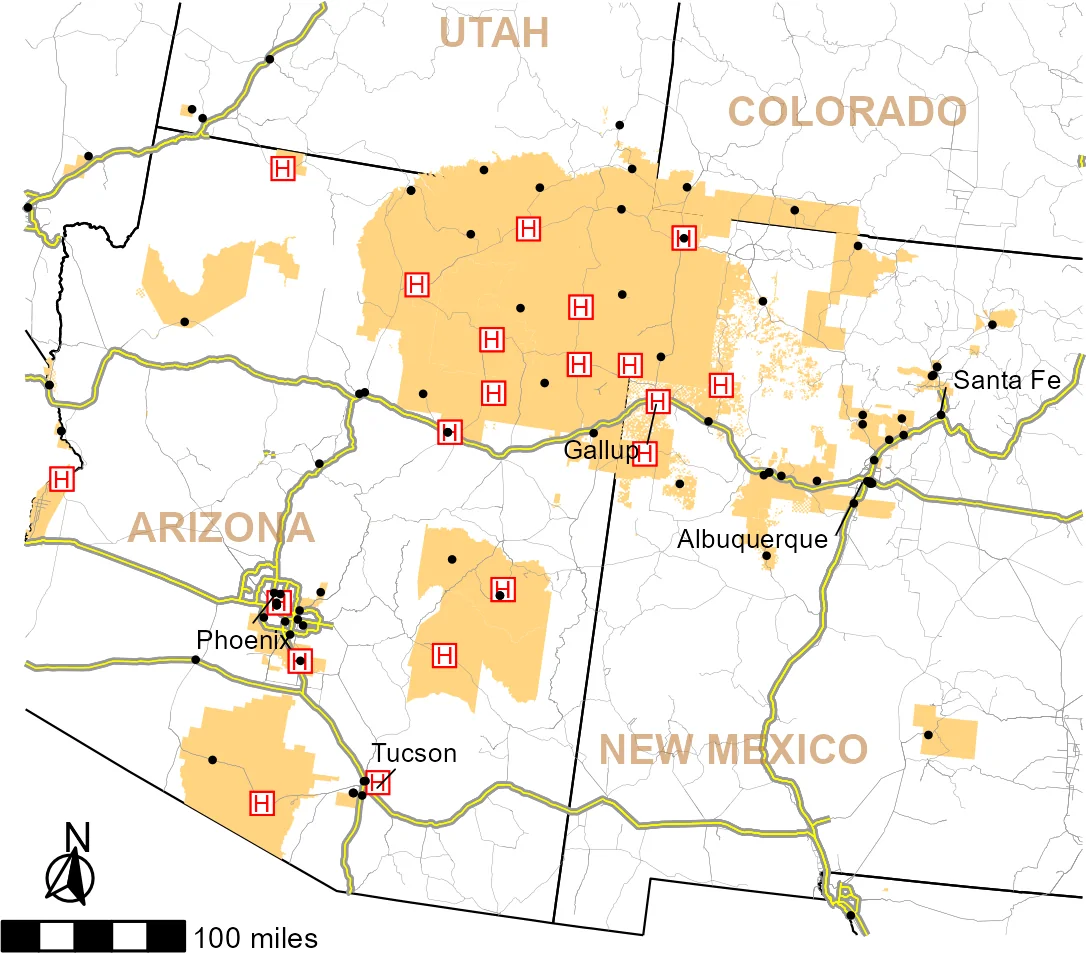
American Southwest tribal lands and the distribution of 119 ambulatory and emergency behavioral health service facilities that may be available to American Indians and Alaska Natives.

**Figure 4.**
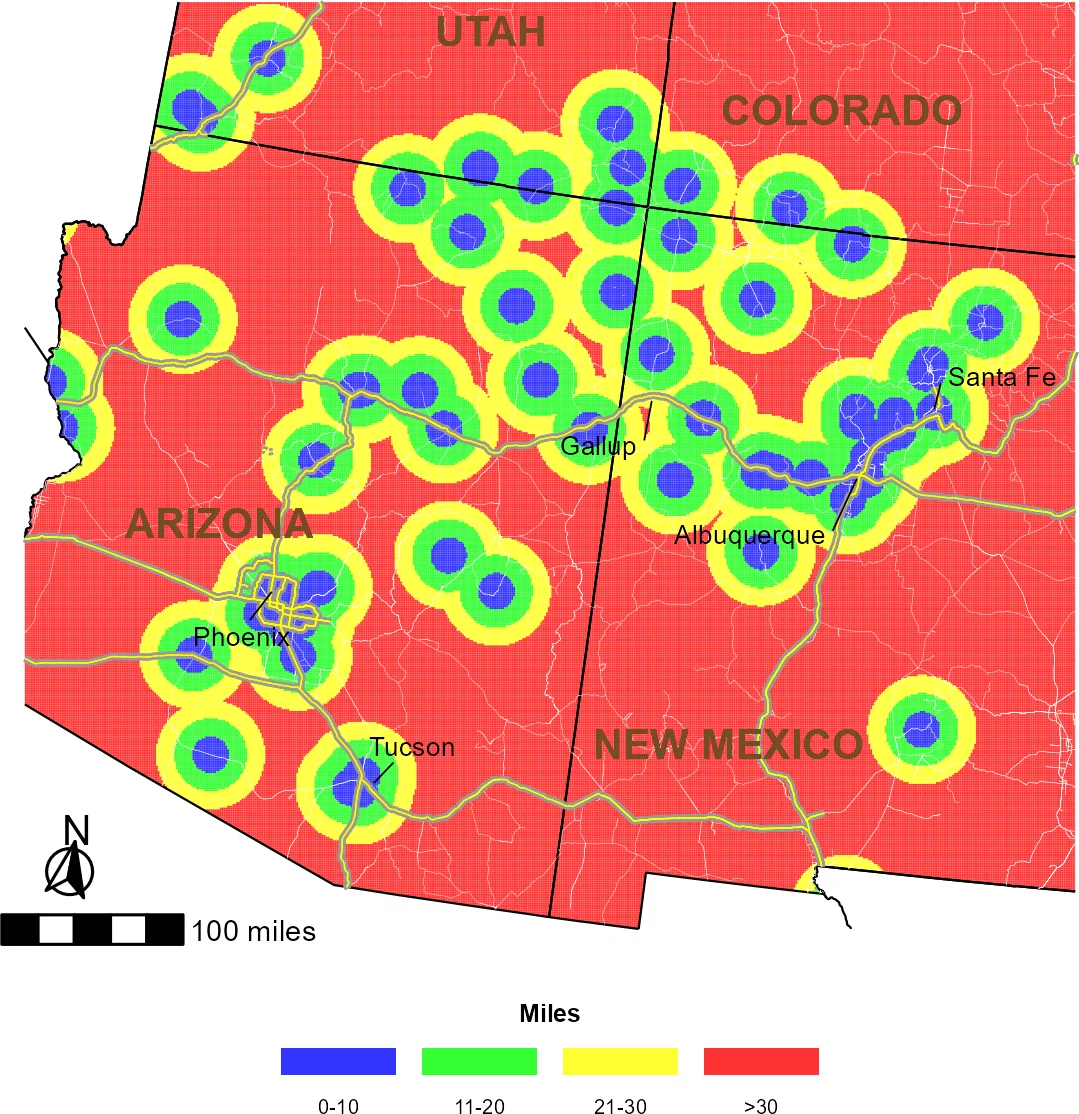
Raster map of American Southwest ambulatory facilities that may be available to American Indians and Alaska Natives, operating during typical business hours (Monday through Friday, 8 AM to 5 PM) and interval straight-line distances away from each facility.

**Figure 5.**
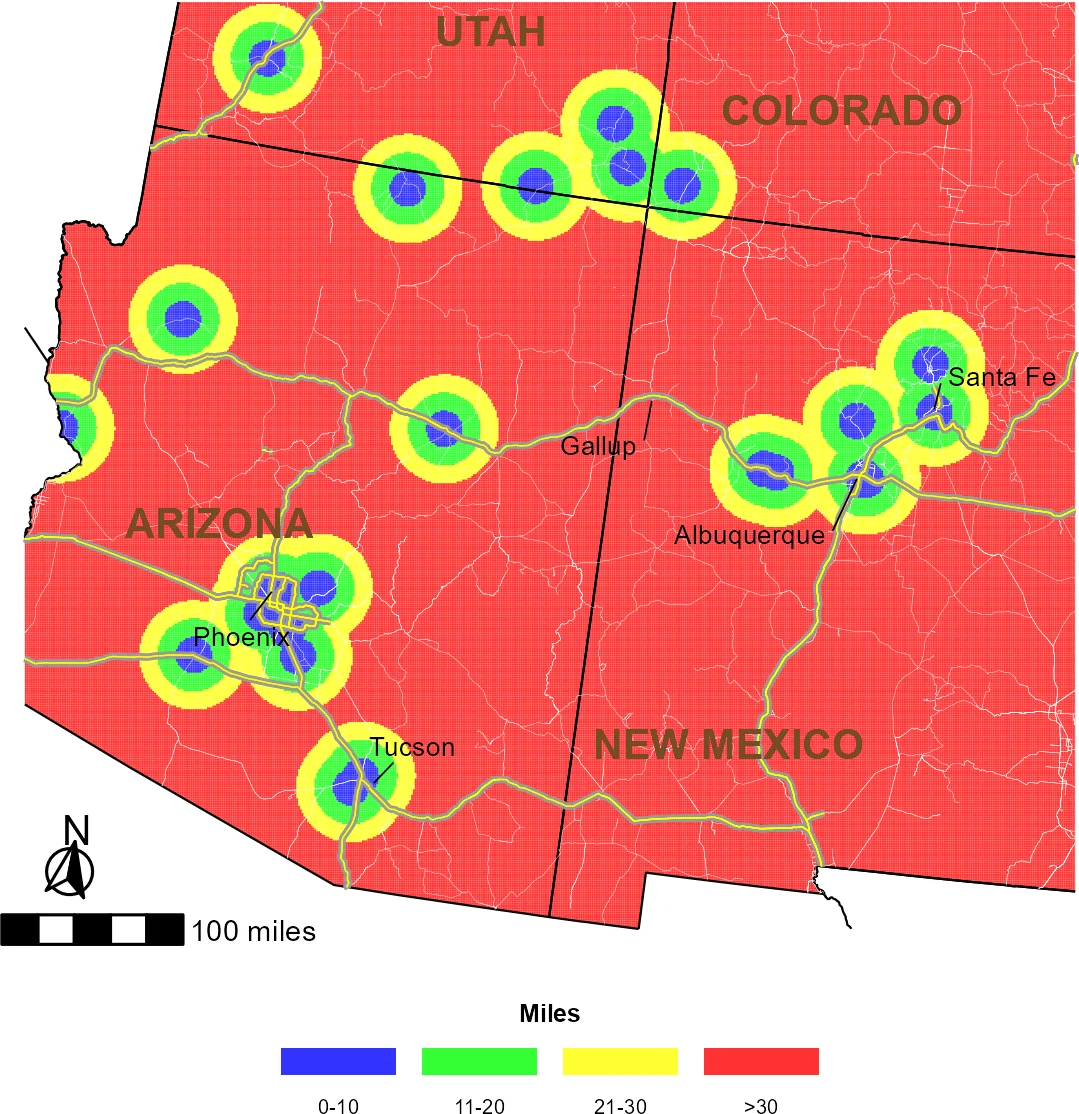
Raster map of American Southwest ambulatory facilities that may be available to American Indians and Alaska Natives, operating during nontypical business hours (Monday through Friday, 5:01 PM to 7:59 AM, plus Saturday and Sunday) and interval straight-line distances away from each facility.

Figure 6 and Figure 7 are maps that illustrate possible LPA areas, relative to distance and facility operating hours. Both maps include a white area that represents tracts that were either beyond 30 miles from available services or were excluded because they fell outside the top quartile by AI/AN population count. This white area includes an estimated 171,311 AI/AN persons, 24.1% of the total regional AI/AN population, which was approximately 710,111 AI/AN persons. The 2 maps illustrate the possible LPA areas for an estimated 538,800 AI/AN persons. Figure 6 illustrates that during typical business hours, an estimated 22,029 (3.1%) AI/AN persons reside in census tracts completely within LPAs, while tracts partially overlapping LPAs contain an estimated 516,771 (72.8%) AI/AN persons. In contrast, Figure 7 illustrates that during nontypical business hours, an estimated 234,852 (33.1%) AI/AN persons reside in census tracts completely within LPAs, while tracts partially overlapping LPAs contain an estimated 303,948 (42.8%) AI/AN persons.

**Figure 6.**
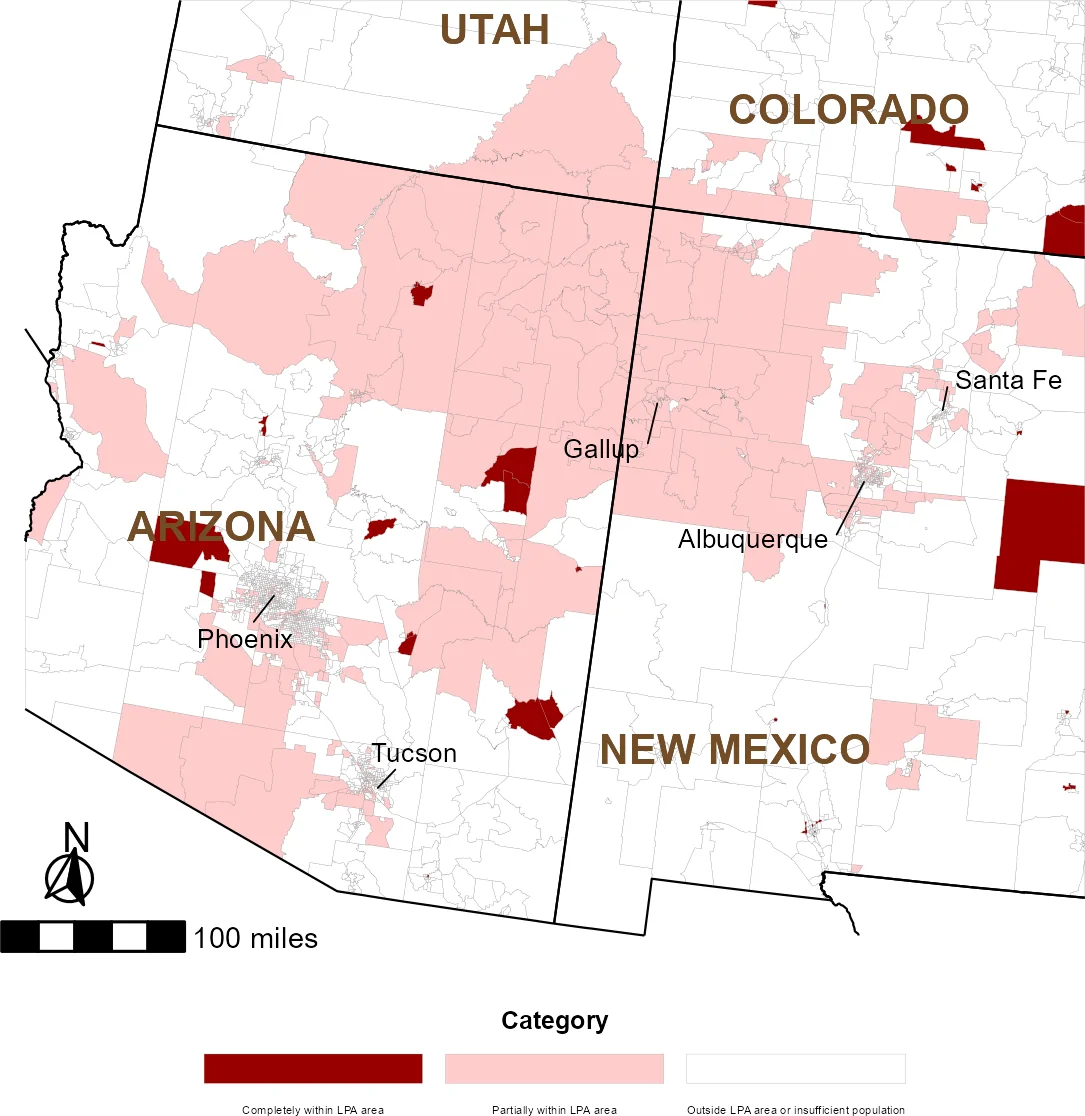
Census tracts in the top quartile by American Indian and Alaska Native population count are classified as completely or partially overlapping low physical access areas during typical business hours (Monday through Friday, 8 AM to 5 PM) for facilities that may be available to American Indians and Alaska Natives.

**Figure 7.**
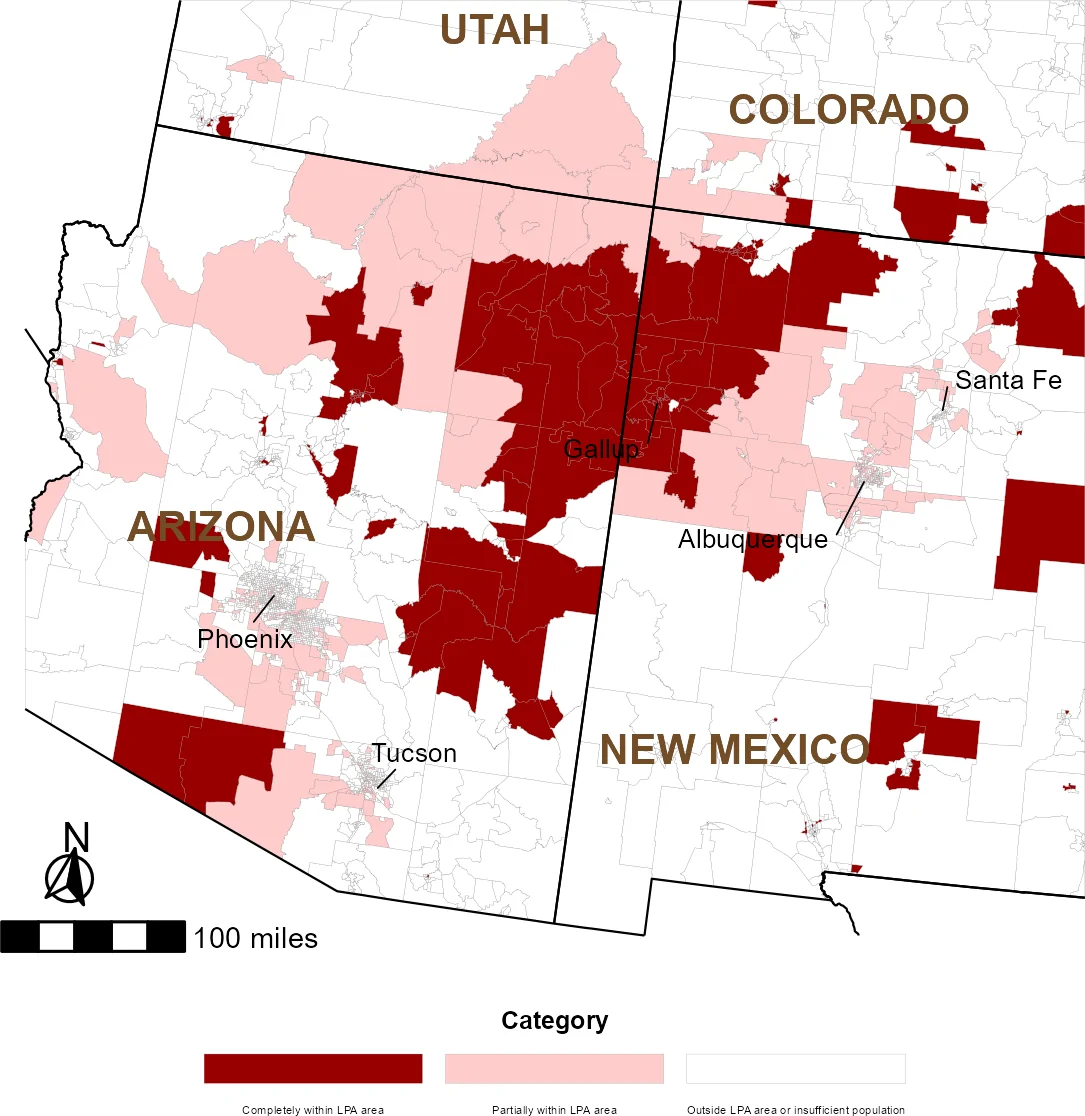
Census tracts in the top quartile by American Indian and Alaska Native population count are classified as completely or partially overlapping low physical access areas during nontypical business hours (Monday through Friday, 5:01 PM to 7:59 AM, plus Saturday and Sunday) for facilities that may be available to American Indians and Alaska Natives.

### Anticipating IRE

As a means for visualizing the hypothesized contextual factors potentially relevant to human trafficking risk, Figure 8 illustrates a 300-mile radius of AI/AN communities surrounding Reno, Nevada. The figure shows how Reno is a hub for a north-south transportation corridor intersecting with east-west interstate highways that span rural counties. The analysis produced a candidate IRE index for prioritizing locations for field validation of hypothesized risk conditions. We calculated an IRE score for each of 80 communities. Of these, 56 (70%) received an IRE score of at least 1.5 and 20 (25%) received an IRE score of 5 or higher. The candidate prioritized surveillance sites are 12 rest stop facilities that were located within 25 km (15.5 miles) of the 80 communities. To protect the identities of tribal communities, their IRE scores are not reported publicly. Likewise, the 12 facilities designated as candidate field-validation locations are not distinguished from the 28 facilities shown herein. Figure 8 illustrates the transportation routes and rest stop facilities (n=28; red squares) that are relevant to the production of IRE indexes and the hypothesized convergence of risk conditions among the 80 AI/AN communities in the study territory.

**Figure 8.**
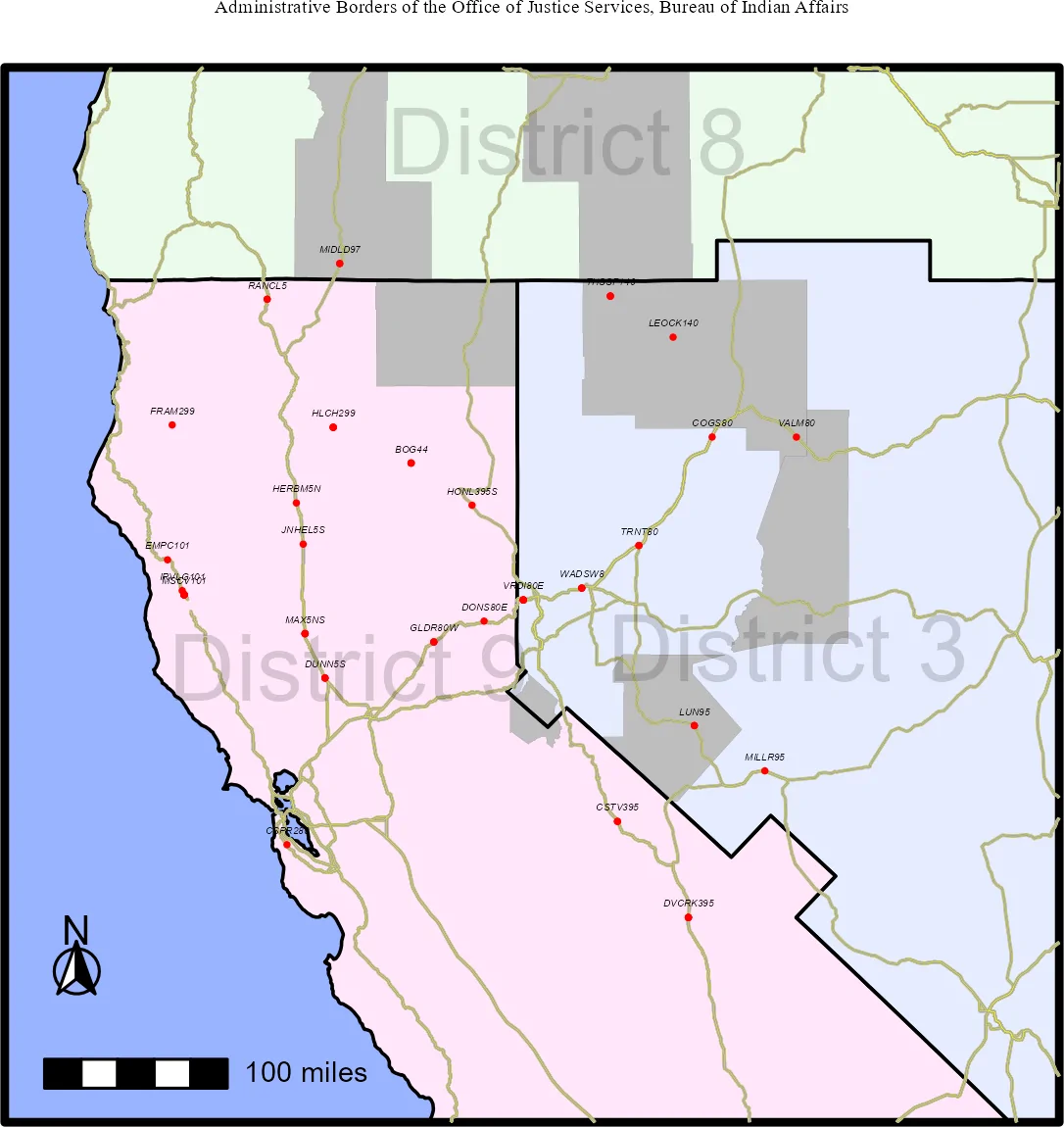
Transportation corridors and 28 rest stop facilities included in the exploratory IRE analysis within a 300-mile radius of Reno, Nevada. Twelve locations met the exploratory candidate field-validation criterion; candidate locations are not distinguished publicly to protect Tribal community identities.

## Discussion

### Principal Findings

The study produced 2 principal findings. First, 3 exploratory geo-analytic frameworks identified candidate priorities for federal, tribal, and regional investigation in 3 domains: elevated and increasing alcohol-related or drug-related mortality, geographic gaps in access to behavioral health services, and locations where hypothesized interpersonal risk conditions could be field-validated. Second, the study demonstrated that routinely updated federal datasets and reproducible GIS methods are technically feasible components of a multicriteria federal decision-support framework. Underlying these findings were 3 sets of study observations. Nationally, 78 counties were identified across 13 states as PIAs. These counties had alcohol-related or drug-related death rates among AI/AN persons at least 25% above the specified national non-AI/AN benchmark, with 6-year pooled crude death rates ranging from 1197.8 to 1848.7 per 100,000. Those 78 counties accounted for 34,783 deaths or 32.2% (34,783/107,954) of the deaths in the analytic sample. In a regional analysis, only 3.1% (22,029/710,111) of the AI/AN population was estimated to be entirely within LPA areas during typical business hours, but this increased to 33.1% (234,852/710,111) during nontypical hours. In another regional analysis, the IRE framework identified 12 candidate field-validation locations from 28 rest stop facilities evaluated within a study region containing 80 AI/AN communities. The factors included transportation corridors, rest stop facilities, and proximity to PIAs.

### Comparison to Prior Work

The PIA analysis provides a repeatable empirical method of organizing and extending the scope of evidence previously developed to support national research on AI/AN behavioral health policy. Etz et al [45] articulated broad principles for the field, emphasizing heterogeneity among Tribal communities, community-oriented approaches to health, and the formation of meaningful partnerships and community benefits. Novins et al [46] operationalized related principles through a national framework that examines geographic samples of tribal communities, service structures, and governance. Luger et al [47] applied similar principles at local and regional scales to develop community-defined research agendas. Collectively, these studies contribute approaches for establishing research priorities, validating local conditions, planning federal initiatives, and developing federal-tribal behavioral health partnerships and plans.

These studies were not designed, however, to provide a routinely updatable analytic mechanism for federal planning across multiple jurisdictions. The PIA analysis is methodologically complementary to this prior work because it provides a reproducible process for identifying geographic areas in which population-level mortality conditions warrant further investigation. In this way, the framework can help organize subsequent cooperative investigations, validation of local conditions, and, where appropriate, negotiations concerning the development of projects or investment priorities.

The PIA framework is also consistent with prior applications of geo-analytics for determining surveillance priorities. Das et al [48] applied spatial analysis to prioritize national sentinel sites for surveillance of ADR medical cases. The PIA methodology applies a related county-level prioritization strategy specifically for AI/AN mortality and demonstrates how sequential policy-relevant thresholds can be incorporated into a broader multicriteria framework. Similarly, the US Environmental Protection Agency used geo-analytics as an initial national decision-support framework for prioritizing local investigations of lead exposure hotspots, where direct surveillance data are limited [49]. Although that analysis focused on identifying hotspots for a specific exposure, the PIA framework demonstrates how multiple morbidity categories can be combined to characterize a broader potential service burden within communities. The PIA approach also illustrates how multiple policy-relevant criteria can be incorporated into the prioritization process, including parameters relevant to federal, state, and local decision-making.

Other investigators have developed multicriteria geographic approaches to health surveillance using vulnerability indices. Daza-Torres et al [50] developed criteria for prioritizing the geographic allocation of wastewater monitoring sites in California. Riggins et al [51] used social vulnerability measures to identify areas in Illinois with elevated priority for preventing sudden unexpected infant deaths. These studies demonstrate the value of reproducible multicriteria geo-analytics for informing recurring government decision-making. Our work extends this general approach by examining multiple analytic domains through geo-analytic frameworks (Figure 1).

The frameworks are designed to permit future geographic integration when federal decisions require intergovernmental or interagency coordination across domains. For example, we identify places where potential service-seeking populations are in LPA areas and areas with conditions for potential IRE. Each of these analyses can function as a unique framework for the respective health domain and can also be integrated with PIA analyses when decisions involve multiple behavioral health risks, service burdens, or resource requirements.

Federal multicriteria geo-analytics may need to combine epidemiological evidence with statutory, administrative, operational, and policy considerations. This is important because the units of federal program decisions are often organizations, jurisdictions, or projects rather than individual patients. Zhan et al [52], for example, used multicriteria geo-analytics in support of prioritizing investments in federally qualified health centers serving populations at high risk for colorectal cancer. Their criteria included policy and program thresholds alongside population-based data. Our LPA analysis follows a similar decision-support logic by combining characteristics of available services, travel distances, and hours of operation, with the geographic distribution of the population of concern, namely AI/AN persons.

Prior research on behavioral health services in AI/AN communities has documented access barriers related to nontypical operating hours, work and family obligations, and transportation arrangements [53]. Our LPA findings are also consistent with research showing that health service access reflects both spatial and nonspatial factors [54]. Operating hours interact with other dynamic dimensions of service accessibility, including staffing, travel conditions, appointment availability, the availability of traditional healing practices, and telehealth and other digital technologies for as-needed access [55-59]. The LPA analysis extends prior federal efforts to characterize LPA to behavioral health services [60] by explicitly incorporating temporal availability into the geographic assessment. The finding that estimated LPA changes substantially between typical and nontypical operating hours illustrates why facility location alone may provide an incomplete representation of service accessibility.

The IRE framework builds on prior geographic and analytic work concerning interpersonal risk, especially in human trafficking [61]. Its underlying spatial logic is consistent with studies examining relationships between proximity, mobility, crime, and victimization. Consistent with prior crime-geography research, the IRE framework incorporates spatial mobility as a hypothesized contextual factor [62]. In contrast to studies that primarily document the prevalence or consequences of victimization among AI/AN persons [63-67], the IRE framework provides a method of identifying locations where hypothesized risk conditions could be investigated and, if validated, where targeted surveillance planning might be considered. The IRE approach is also consistent with recommendations for interagency coordination of risk information involving transportation, public safety, and tribal jurisdictions [68]. If validated through field research, the framework could support federally funded studies and federal-tribal partnerships aimed at preventing human trafficking, consistent with concerns and priorities previously raised by tribal researchers and leaders [69,70].

### Limitations

Several limitations are common to all 3 frameworks. The parameter thresholds and weights used in these exploratory analyses have not been validated, the analyses rely primarily on secondary public datasets, and the resulting measures support area-level rather than individual-level inference. The analyses do not incorporate potentially important covariates, such as workforce availability and service capacity, that may influence future algorithmic designs [71].

Accordingly, the PIAs, LPAs, and IRE locations should be interpreted as priorities for further investigation rather than as validated determinations of community-level or individual-level risk. All 3 frameworks will require sensitivity testing and validation with tribal and local partners before operational use.

In addition, the PIA analysis identifies worsening ADR mortality from 2018 to 2023, a period that overlaps substantially with the COVID-19 pandemic. Pandemic-related changes in mortality, service availability, social conditions, and use of health services may therefore have influenced the observed geographic patterns. Future analyses could incorporate additional indicators, such as the prevalence of active substance use disorders and geographically associated criminal or behavioral events, to better characterize local risk conditions. However, the use of such data would raise individual privacy, tribal data sovereignty, and governance considerations requiring specialized protection and appropriate tribal consultation. The PIA analysis is also limited by its reliance on mortality data. County-level estimates may be unstable when AI/AN population denominators or death counts are small, and misclassification of AI/AN race on death certificates may bias mortality estimates. In addition, the state-level decision thresholds may exclude small-population jurisdictions that nevertheless experience high mortality rates.

The LPA analysis is limited by its use of straight-line distance as an approximation of physical access. This measure does not account for road networks, terrain, seasonal conditions, or other geographic factors that can affect travel time. The analysis also does not incorporate several operational characteristics of facilities, including workforce availability, appointment capacity, or variation in service levels. Incorporating road-specific travel times and locally validated operational data could improve the precision and geographic applicability of future LPA analyses. Facility-specific eligibility may affect access to services, despite their locations being in proximity to AI/AN persons. For tracts partially overlapping with LPAs, population counts describe the tract population and should not be interpreted as the number of individuals residing specifically within the low-access portion of the tract.

The IRE analysis is the most exploratory of the 3 frameworks. Although it may be sufficiently developed to inform initial interagency and tribal field-validation studies, it is not yet appropriate for scalable policy, predictive, or operational use. Its parameters and weights are theoretical and require empirical validation. Future analyses will also require localized data to characterize interpersonal risk conditions, mobility patterns, and the prevalence and distribution of injurious events. The state-level Hotline data used to select the demonstration region represent reported and identified cases rather than population-based estimates of trafficking incidence; geographic differences in reporting or identification may therefore affect comparisons among states.

### Conclusions

The 3 geo-analytic frameworks demonstrate complementary methods for identifying geographic priorities for the investigation of behavioral health risks and service conditions among AI/AN populations within overlapping tribal and rural health service environments. The PIA analysis provides a repeatable method for identifying areas where observed mortality patterns warrant further investigation and local validation. Its national application also illustrates opportunities for cooperative investigations across jurisdictions, including refinement of measures in which small population denominators may affect estimates.

The LPA analyses demonstrate how geographic access measures can incorporate both the location and temporal availability of services. In the American Southwest, the estimated proportion of the AI/AN population residing completely within LPA areas increased from 3.1% (22,029/710,111) during typical operating hours to 33.1% (234,852/710,111) during nontypical hours. This difference suggests that measures based solely on facility location may substantially understate physical access constraints when operating hours are not considered. LPA analyses can therefore help identify potential service gaps that warrant further investigation and may inform subsequent service planning and negotiations.

The IRE framework provides a third, more exploratory application by identifying locations where hypothesized interpersonal risk conditions may warrant field investigation. Rather than predicting interpersonal violence or trafficking events, the framework demonstrates how multiple geographically coincident conditions can be organized to prioritize locations for validation and potential surveillance through interagency and federal-Tribal cooperation.

Taken together, the PIA, LPA, and IRE analyses demonstrate the methodological feasibility of a reproducible, multicriteria geo-analytic approach to federal decision support. Each framework addresses a distinct domain, and the analyses are designed to permit future geographic integration when decisions involve multiple behavioral health risks, service-access constraints, jurisdictional interests, or resource considerations. Because the methods use routinely updated federal datasets or datasets supervised by federal agencies and repeatable GIS procedures, they can provide a basis for iterative analyses in which policy-relevant parameters can be modified, tested, and reviewed as conditions change.

Such reproducible methods may be particularly useful for federal decisions that require coordination among agencies [72], Tribal governments, state and local health systems, and other partners. Federal policy already emphasizes improved data governance, program evaluation, and interoperability of geographic information [73,74]. A common geo-analytic framework could contribute to these objectives by providing a transparent evidentiary basis for identifying priorities, comparing conditions across geographic scales, organizing cooperative investigations, and forming geo-analytic communities of practice [75]. Longer-term development could incorporate more dynamic data and GeoAI capabilities to support increasingly complex and repeatable place-based analyses through shared services [76,77].

Standardization of analytic methods, however, should not imply uniform selection of parameters across tribal communities. Field validation, sensitivity testing, tribal consultation, and, where appropriate, tribal approval or codevelopment are necessary before these frameworks are used for operational decisions. Federal decision-support methods should therefore provide a common analytic structure while allowing parameters and interpretations to reflect locally relevant conditions, governance requirements, and tribal priorities.

This study demonstrates the feasibility of using routinely available federal data and reproducible GIS methods to identify geographic priorities for investigating behavioral health risk, service accessibility, and hypothesized interpersonal risk conditions among AI/AN populations. With appropriate validation and tribal and local engagement, these exploratory frameworks could contribute to a common evidentiary foundation for federal-tribal research, interagency planning, and multicriteria decision support across complex jurisdictional settings and federal service domains [77].

## Abbreviations

ADR: alcohol-related and drug-related
AI/AN: American Indian or Alaska Native
CDC: Centers for Disease Control and Prevention
EO: executive order
GeoAI: Geospatial AI
GIS: geographic information systems
HHS: Health and Human Services
*ICD-10*: *International Classification of Disease, Tenth Revision*
IHS: Indian Health Service
IRE: interpersonal risk exposure
LPA: low physical access
PIA: prioritized investigation area
WONDER: Wide-Ranging Online Data for Epidemiological Research
